# Deciphering the Psychological Characteristics of Takotsubo Cardiomyopathy and Acute Myocardial Infarction

**DOI:** 10.3390/jpm15010006

**Published:** 2024-12-27

**Authors:** Maria Casagrande, Giuseppe Forte, Francesca Favieri, Alessandro Mingarelli, Francesca Agostini, Luca Arcari, Ilaria Passaseo, Raffaella Semeraro, Giovanni Camastra, Viviana Langher, Luca Cacciotti

**Affiliations:** 1 Dipartimento di Psicologia Dinamica, Clinica e Salute, Università di Roma “Sapienza”, Via Degli Apuli 1, 00185 Roma, Italy; g.forte@uniroma1.it (G.F.); francesca.favieri@uniroma1.it (F.F.); alessandro.mingarelli@psicosalute.com (A.M.); viviana.langher@uniroma1.it (V.L.); 2 Dipartimento di Psicologia, Università di Roma “Sapienza”, Via dei Marsi 78, 00185 Roma, Italy; francesca.agostini@uniroma1.it; 3 UOC di Cardiologia, Ospedale M.G. Vannini, 00189 Roma, Italy; luca.arcari@uniroma1.it (L.A.); ilaria.passaseo@gmail.com (I.P.); semerella.s@libero.it (R.S.); gcamastra@virgilio.it (G.C.); 4 Divisione di Cardiologia, Policlinico Casilino, Via Casilina, 00169 Roma, Italy; cacciotti@figliesancamillo.it

**Keywords:** takotsubo syndrome, acute myocardial infarction, psychopathological symptoms, distress, perceived social support, health locus of control

## Abstract

**Background/Objectives**: Takotsubo syndrome (TTS) shares many clinical features with acute myocardial infarction (AMI); however, its underlying pathophysiology remains elusive due to specific characteristics (i.e., reversibility, presence of stressors, and low mortality rate). Emerging evidence suggests that TTS often emerges following significant somatic, emotional, or psychological stressors. Several studies have investigated the biological factors that may contribute to the onset of TTS, including the release of stress hormones. However, the psychological characteristics associated with TTS, which may be useful for differentiating TTS from other cardiovascular conditions, remain poorly explored. This study aims to overcome these limitations by evaluating whether certain psychological features may allow this specific clinical condition to be differentiated from other cardiovascular conditions. **Methods**: This study compared three groups of individuals: patients with TTS (N = 56), patients with AMI (N = 52), and individuals without a history of cardiovascular disease (N = 56). Patients were examined three months after the acute phases. The objective was to identify and examine multiple psychological factors involved in TTS, including state and trait anxiety, symptoms of depression, psychological symptoms, distress, perceived social support, and health locus of control. **Results**: The results indicate that patients with TTS exhibited higher levels of somatization, trait anxiety, obsessive-compulsiveness, and overall psychological distress than patients with AMI and control groups. These findings were associated with an internal health locus of control and low-risk lifestyles. **Conclusions**: This psychological exploration of TTS reveals a multifaceted relationship between the mind and the heart, challenging traditional notions of cardiovascular pathophysiology and suggesting the importance of considering the cardio-psychological health of patients in both preventive and intervention programs.

## 1. Introduction

The mind–heart interaction has been confirmed by the many studies that have explored the relationship between psychological aspects and cardiovascular diseases (e.g., [1]. Nevertheless, many questions remain unanswered, despite the growing body of evidence supporting psycho-cardiovascular interactions. While the role of mental health on cardiovascular health and vice versa has been highlighted, there is still a paucity of evidence regarding the psychological characteristics that may help in defining different heart conditions. Given these considerations, [2] may offer valuable insights for studying the takotsubo syndrome (TTS; “stress cardiomyopathy”). TTS is an acute coronary syndrome characterized by severe left ventricular (LV) dysfunction that typically recovers spontaneously in the absence of obstructive coronary artery disease and permanent structural damage. This distinguishes it from acute myocardial infarction, which has a higher mortality rate [3,4]. The most recent epidemiological data indicate that TTS has a prevalence of 2% in the general population, with an incidence of hospitalization due to TTS ranging from 2.3 to 7.1 per 100,000 persons per year in a comparison between 2007 and 2012 [5]. Although the etiology, epidemiology, and pathophysiology of TTS remain partially unknown, evidence suggests that stress and the subsequent cascade of catecholamines play a significant role in its pathogenesis [5,6]. Clinical observations have confirmed the frequent occurrence of emotional or physical events perceived as stressful and preceding the onset of TTS. Some authors have proposed that the way individuals respond to the precipitating factors may influence the prognosis of TTS [7,8]. The subjective component of stress perception is influenced by individual and psychological characteristics. This may represent a discriminant factor between TTS and other cardiovascular conditions. Consequently, higher anxiety and symptoms of depressions than those observed in Acute Coronary Syndrome or AMI [9,10,11] and higher levels of health and well-being concern in the recovery phase among patients with TTS would be some of the psychological characteristics of TTS. To gain a deeper understanding of these aspects and to interpret them within the context of the psycho-cardiovascular perspective, it is essential to identify potential risk factors for symptom exacerbation, recurrence, or long-term negative effects on general health. A comparison between TTS and other cardiac diseases is therefore necessary.

A review of the literature identifies three core psychological constructs that may serve as potential transdiagnostic predictors of more effective management of a cardiovascular event. These include general psychopathological symptomatology, with a particular focus on symptoms of depression and anxiety, locus of control and perceived support. The term “general psychological distress” refers to the emotional suffering that is characterized by different signs and symptoms related to psychological problems, including symptoms of depression and anxiety. Psychological distress has been identified as a significant risk factor for the onset and progression of coronary artery disease (CAD) [12]. In approximately one-fifth of individuals with stable CAD, acute mental stress was reported [13]. The term “perceived support” refers to an individual’s cognitive appraisal of feeling connected and supported by others. Previous studies have indicated that perceived social support was strongly associated with the risk and management of cardiovascular diseases [14]. Finally, locus of control refers to the belief regarding the extent to which one’s health is within one’s control (internal control) or not (external control). LOC was defined as a key variable to intervene on well-being of cardiovascular disease patients [15,16]. Our study aimed to compare patients with a diagnosis of TTS with both patients with AMI and controls who did not report a history of cardiovascular pathologies to understand the role of these psychological variables after the onset of cardiovascular disease.

Since TTS typically mimics the physical symptoms of acute myocardial infarction (AMI), but differences were reported in psychological symptoms [17], a comparison between these two syndromes is useful for better understanding the relationship between psychological aspects and cardiovascular disease. The main hypothesis, in line with the findings of previous studies [18,19,20,21,22,23,24,25] was to report a generally worse mental condition in patients after the experience of cardiac pathology than in healthy controls, as well as higher general risk factors for cardiovascular health (e.g., lifestyles, habits, familiarity, clinical history). Moreover, given the differences between TTS and AMI in terms of etiology and prognosis, and considering the mind–heart hypothesis, we assumed that there would be a greater prevalence of anxiety and symptoms of depression in TTS than in AMI. Furthermore, we predicted that there would be a greater number of psychopathological symptoms and a greater number of risk-related dimensions (such as low perceived social support) in TTS than in AMI. To gain insight into the relationship between cardiovascular and mental health, we also aimed to examine whether personal characteristics and psychological outcomes were associated with TTS and AMI diagnosis. Finally, to ensure the results are as generalizable as possible, and to overcome the limitations of previous studies, the analyses were controlled for several demographic variables that may potentially influence the results, including sex.

## 2. Materials and Methods

### 2.1. Participants

Three groups of participants were recruited, resulting in a total of 164 individuals (56 TTS; 52 AMI; 56 CG). All participants were recruited at the same cardiology clinic between 2016 and 2018. Enrollment for AMI and TTS was conducted at least 3 months after the cardiovascular event (i.e., chronic phase of the disease). TTS diagnosis was performed according to the following criteria: (1) acute onset of symptoms, (2) absence of a culprit lesion on coronary angiography, (3) typical apical ballooning, (4) elevated cardiac biomarkers, and (5) normalized left ventricular systolic function on follow-up echocardiography [26]. AMI diagnosis following subsequent criteria: (1) acute onset of symptoms, (2) elevated cardiac biomarkers, (3) a diagnosis of myocardial infarction confirmed by a cardiologist, via coronary angiography and clinical evaluation, and consequent hospitalization. For CG, inclusion criteria were (1) the absence of any heart disease in clinical history and (2) an age range like that of the two groups of patients. The control group was enrolled via a snowball sampling technique, whereby patients requested the enrollment of relatives or friends. The main characteristics of the groups are shown in Table 1 and Table 2.

### 2.2. Instruments

Physiological measures

Systolic (SBP) and diastolic blood pressure (DBP) and heart rate (HR) were recorded using an automatic electronic sphygmomanometer, according to the European guidelines criteria [27]. Participants were seated on a comfortable chair with their backs resting on their backrests. Following a 5 min rest period, participants were instructed to remain silent while the three measurements were taken on the non-dominant arm, with a 2 min interval between each measurement. The mean values of both SBP and DBP were considered for subsequent analyses. Weight and height were used to calculate the Body Mass Index (BMI), dividing weight (in kilograms) by height (in meters squared) [28].

Sociodemographic information

A face-to-face interview was conducted to collect data on the participants’ sociodemographic characteristics (age, education, occupation, marital status). Anamnestic information regarding their medical history, including any psychiatric consultation or psychological treatment, and the presence of potential risk factors for cardiovascular diseases were also investigated. Specifically, information about medical conditions associated with higher cardiac risk (i.e., hypertension, hypercholesterolemia, hyperglycemia) was collected. Then, subjects’ lifestyle habits were investigated, including their smoking habits (number of cigarettes smoked per day), alcohol consumption (number of glasses of wine/beer consumed per day), caffeine consumption (number of cups of coffee consumed per day), and exercise habits (yes/no). Furthermore, a list was compiled of all pharmacological treatments that the subjects were currently undergoing.

Psychological measures

State-Trait Anxiety Inventory (STAI; [29,30]). The STAI is a self-evaluation scale designed to assess both state and trait anxiety. It consists of a 40-item questionnaire, divided into 20 items that assess state anxiety (STAI-S), which is defined as the subjective experience of anxiety at the moment” and 20 items that assess trait anxiety (STAI-T), which is defined as the general disposition towards anxiety. Responses are indicated on a four-point Likert scale, ranging from 1 (not at all) to 4 (very much so), resulting in total scores from 20 to 80, where higher scores indicate greater anxiety [31]. The internal consistency coefficients of the adopted version ranged from 0.86 to 0.95.

The 90-item Symptom Check List (SCL-90; [32,33]). The SCL-90 is a psychometric instrument designed to assess psychological distress. Each of the 90 items is rated on a five-point Likert scale, ranging from ‘not at all’ (0) to ‘extremely’ (4). Subsequently, the answers are combined in nine primary symptom dimensions: somatization, obsessive-compulsive, interpersonal sensitivity, depression, anxiety, hostility, phobic anxiety, paranoid ideation, and psychoticism. in addition, three global indices provide measures of overall psychological distress: the Global Severity Index, the Positive Symptom Total, and the Positive Symptom Distress Index. The SCL-90 specifically measures the symptomatology in the past 7 days. The psychometric properties of this scale have been well established by numerous studies. The internal consistency coefficient Cronbach alpha of the Italian validation for the nine subscales ranged from 0.77 to 0.90.

Multidimensional Scale of Perceived Support (MSPS; [34,35]). The MSPS is a self-report instrument designed to assess an individual’s perceived social support. It is a multidimensional construct that refers to an individual’s perception of being part of a supportive relationship network. The MSPS is a 12-item self-administered test that employs a 7-point Likert scale. The level of perceived social support is assessed on three subscales: family, friends, and significant other. The Italian adaptation [35] reports psychometric properties data, detecting a good internal consistency of the instrument with a Cronbach alpha of 0.88.

Beck Depression Inventory (BDI; [36,37]). The BDI is a self-assessment questionnaire composed of 21 multiple-choice items that evaluate the behavioral manifestations of depression over the two weeks preceding the administration date. The questionnaire items assess the severity of the symptoms characterizing the depressive disorder. It focuses on the cognitive–affective and somatic–vegetative dimensions of depression. For each item, participants must indicate a score ranging from 0 to 3 (total range 0–63), selecting the option that best reflects their condition. A final score of between 0 and 9 indicates the absence of depression, a score between 10 and 18 designates a mild level of depression, scores between 19 and 29 points indicate a moderate level of depression, and a score between 30 and 63 indicates a severe level of depression. The internal consistency of the instrument is about 0.90.

Health Locus of Control Scale (HLC; [38,39]). The HLC was designed to measure health-specific control expectations to predict health-related behavior. The HLC scale includes 11 items, with 6 items phrased in an external direction and 5 items phrased in an internal direction. The scale has a potential scoring range from 11 (internal HLC) to 66 (external HLC). Therefore, lower scores indicate internal HLC, while higher scores indicate external HLC. Individuals with external HLC believe that their health is determined by external factors beyond their control, such as fate, luck, or chance events. Conversely, individuals with internal HLC believe that a person’s behavior plays a crucial role in determining their health or illness.

### 2.3. Procedure

Following the signing of the informed consent form, the participants were subjected to blood pressure recordings and measurements of their weight and height. Subsequently, the participants underwent the socio-demographic and anamnestic interview and completed the questionnaires. The procedure lasted 40 min and was conducted in a quiet environment with a comfortable temperature. The research was conducted according to the Declaration of Helsinki principles. The Ethics Committee of Dynamic and Clinical Psychology and Health Studies, of “Sapienza” University of Rome approved it (Prot. n. 0000664).

### 2.4. Data Analysis

Continuous variables were expressed as means and standard deviations (SD), while categorical variables were expressed as numbers (N) and percentages (%). To compare categorical variables, the χ^2^ test was used. Specifically, marital status, occupational status, and some cardiac risk factors (smoking habits, diabetes, hypertension, hypercholesterolemia, family history of cardiovascular diseases) were tested. To compare continuous variables, Analysis of Variance (ANOVA) and Covariance (ANCOVA) were carried out to control for the differences between the three groups (CG, TTS, AMI). Specifically, age, years of education, some cardiac risk factors (BMI, number of cigarettes), clinical recordings (SBP, DBP, HR), lifestyles and psychological variables were tested. To control for the potentially confounding role of sex, age, years of education, body mass index, and smoking, these variables were considered as covariates. The planned comparisons via orthogonal contrasts were adopted to verify the differences between the groups by contrasting the means of each group.

A multinomial logistic regression model was employed to identify variables associated with cardiovascular disease. Variables introduced in the model were sex, age, and all psychological variables that were measured. In all analyses, the significance level was defined at *p* < 0.02 according to Bonferroni’s correction. Statistical analyses were performed using Jamovi.

## 3. Results

### 3.1. Demographics, Lifestyles, and Physiological Variables

The socio-demographic and clinical characteristics of the sample are shown in Table 1. Groups were significantly different regarding sex (*p* < 0.0001), age (*p* < 0.005; ƞp^2^ = 0.06), years of education (*p* < 0.01; ƞp^2^ = 0.05), BMI (*p* < 0.03; ƞp^2^ = 0.04), the number of cigarettes smoked per day (*p* < 0.02; ƞp^2^ = 0.04), marital status (; *p* < 0.004), caffeine intake (*p* < 0.001; ƞp^2^ = 0.08), employment (χ^2^ = *p* < 0.006), family history of cardiovascular diseases (*p* < 0.03), systolic blood pressure (*p* < 0.001; ƞp^2^ = 0.001), presence of hypertension (*p* < 0.001), and medications (beta-adrenergic blocking agents, ACE inhibitors, antiplatelet medications, and lipid-lowering drugs) (*p* < 0.001). Other variables were comparable between groups (Table 1). Planned comparison evidenced that patients with TTS were older, less educated, and had lower BMI compared to both CG (age: *p* < 0.01; BMI: *p* < 0.02; years of education: *p* < 0.01) and AMI (age: *p* < 0.003; BMI: *p* < 0.01; years of education: *p* < 0.005). No difference was observed between AMI and CG (age: *p* = 0.69, BMI: *p* = 0.81; years of education: *p* = 0.77). Moreover, AMI smoked fewer cigarettes per day (*p* < 0.005) than TTS and consumed more caffeine than TTS (*p* < 0.05) and CG (*p* < 0.04) (Table 1). Clinical recordings showed higher systolic blood pressure in TTS than in CG (*p* < 0.05) and AMI (*p* < 0.0002), and IMA and CG were different (*p* < 0.05). Heart rate measures were significantly lower in TTS compared to CG (*p* < 0.02) TTS (χ^2^ = 15.9; *p* < 0.001) and AMI (χ^2^ = 14.1; *p* < 0.001). The prevalence of smoking habits among TTS patients is significantly lower than that among CG (χ^2^ = 3.61; *p* < 0.05) and AMI patients (χ^2^ = 5.91; *p* < 0.01). About pharmacological treatment, TTS (χ^2^ = 15.5; *p* < 0.001) and AMI patients (χ^2^ = 15.0; *p* < 0.001) use more drugs than CG. Finally, AMI patients exhibited a higher prevalence of a familiar history of cardiovascular disease compared to those with TTS (χ^2^ = 6.63; *p* < 0.01). No other significant differences were found.

### 3.2. Psychological Aspects

Psychological dimensions adjusted for covariates of the study sample are shown in Table 2. The ANOVA on psychological variables reported some significant between-group differences. Considering psychological symptoms assessed via SCL-90, TTS patients reported higher somatization and phobic anxiety than both AMI patients and CG (all *p* < 0.01). In contrast, in obsessive-compulsive, depressive symptoms, and global symptomatology, TTS patients reported higher scores than AMI patients (*p* < 0.01) but not higher scores than CG (see Table 2). BDI score was higher in TTS patients than CG (*p* = 0.02), but no statistical differences emerged between AMI patients and both TTS patients and CG. ANOVA revealed a more internal health locus of control in TTS than in both AMI and CG (*p* < 0.01). The results on perceived social support indicated that TTS exhibited lower levels of perceived support from friends than CG (*p* = 0.006), while no other significant differences were observed. Moreover, ANCOVAs were conducted to control for demographic and lifestyle variables that significantly differed between the groups (age, years of education, BMI, smoking habits), particularly for sex. These analyses did not confirm all the significant differences.

### 3.3. Multinomial Logistic Regression

To verify the potential predictive role of the psychological variables while controlling for sex differences, a hierarchical model of multinomial logistic regression was tested. In the first block, the influence of sex and age in the group condition was examined (Model 1; R2McF = 0.21; *p* < 0.0001). A second tested the combined effect of sex and the set of psychological variables (all the SCL-90 subscales, trait and state anxiety assessed via STAI, BDI score, HLC index, and the subscales of the MSPSS; Model 2: R McF 2 = 0.47; *p* < 0.0001). The comparison of the two models revealed a significant increase in the fit index (χ^2^ = 95.7; *p* < 0.0001). Specifically, considering Model 2, the odd risk ratio of belonging to the TTS group compared to the CG group was found to be significantly higher for females. Additionally, a low score on the friend subscale of the MSPSS, a higher score on state anxiety, sensitiveness, psychoticism, anxiety, and an internal locus of control were associated with an increased likelihood of belonging to the TTS group (all *p* < 0.05; see Table 3). The odd risk ratio for TTS compared to AMI was significantly predicted for the female sex, a lower score in the friend subscale of the MSPSS, higher scores in anxiety and hostility assessed via SCL-90, and internal locus of control (all *p* < 0.05; see Table 3). Regarding the odd ratio for AMI compared to CG, higher BDI scores and lower family support were associated with diagnosis (see Table 3).

## 4. Discussion

Takotsubo syndrome and acute myocardial infarction represent distinct cardiac pathologies yet share symptomatic overlaps. This paper explores the role of psychological factors in distinguishing TTS from AMI with a focus on diagnostic challenges and potential therapeutic implications. Through a comprehensive evaluation of psychological aspects, this study synthesizes evidence on psychological aspects, examining their differential association with TTS and AMI diseases.

Since its initial definition, TTS has been identified as an acute heart failure syndrome triggered by a negative or stressful physical or emotional event [18,19,20,21,22,23,24,25].

Subsequently, it has been recognized that the stressful events need not necessarily be negative [40,41], suggesting a role for emotionally salient experiences per se [8,20]. As indicated by our previous findings, the frequency and objective impact of a life event did not discriminate TTS from other conditions. However, the subjective interpretation of the events played a role in the occurrence of TTS. Moreover, the higher frequency of emotional triggers [8] suggests that TTS could present certain personality patterns that may shape individuals’ vulnerability to some stimuli (e.g., external events) that could be then interpreted as salient (i.e., stressful) more frequently, despite other populations with different personality patterns. However, little attention has been paid to analyzing these aspects in individuals with TTS and how they differ from other heart failure conditions.

This study investigated this pattern, focusing on a range of psychological characteristics potentially associated with TTS and AMI, in contrast to previous studies that analyzed single psychological aspects [20,21,22,23,24,25]. Our results indicated that patients with TTS exhibited higher somatization, trait anxiety, obsessive-compulsive symptoms, and overall psychological distress compared to those with AMI. Previous studies have demonstrated that after the cardiovascular event TTS [23,24] and other ‘atypical’ cardiac diseases [42,43] are associated with anxious responses [42].

Anxiety and heart diseases appear to be associated in a bidirectional relationship, with each condition exacerbating the other. Acute episodes of anxiety, such as panic attacks, can trigger physiological responses, including sympathetic nervous system activation and increased catecholamine release, which may precipitate cardiac health. Conversely, the diagnosis of heart disease or experiencing cardiac events can significantly increase anxiety levels. This heightened anxiety may further exacerbate cardiovascular risk by promoting maladaptive behaviors such as smoking, a sedentary lifestyle, and poor medication adherence [44,45]. The psychological pattern of patients with TTS, characterized by anxiety and other dysfunctional patterns, appears to be consistent with an emotional dysregulation that can be associated with the presence of systolic hypertension in them. This result is consistent with the known association between heart-related conditions and emotional dysregulation, including early and less severe conditions, such as essential blood hypertension (e.g., [46,47]). However, the high anxiety and dysfunctional psychological characteristics observed in patients with TTS could also be attributed to the sex imbalance of the sample, given the higher prevalence of these psychological disorders in female individuals compared to male individuals (e.g., [48,49]).

In contrast to previous studies [9,10,11], we did not observe higher symptoms of depression in patients with TTS. Symptoms of depression in the TTS group were comparable to those reported by patients with AMI and healthy individuals. These results are likely due to some methodological aspects, such as the lack of control for confounding variables (such as age) that could have influenced the relationship. This hypothesis was confirmed by the analysis without covariates, which reported a statistical difference between TTS and CG that disappeared when covariates were introduced in the analysis, demonstrating that age may partially mediate the relationship between depressive symptoms and clinical conditions of TTS.

Higher somatization, intrusive thoughts, and an internal health locus of control complete the dispositional pattern of patients with TTS compared to those diagnosed with AMI. Individuals with an internal LOC have a reduced cortisol response to a stressor when they perceive themselves as having some control but a higher response when they perceive no control, such as during stressful and unexpected events [50]. Moreover, individuals with an internal LOC are more likely to engage in positive health behaviors. An analysis of sociodemographic variables confirmed this result. Accordingly, patients with TTS are characterized by a difficulty to control some stressful situations that could define a specific individual response associated with a cardiovascular event.

Patients with TTS exhibited higher levels of somatization and intrusive thoughts than other groups. Higher levels of somatization and obsessive-compulsive symptoms are consistent with previous observations made in patients with TTS [20,23,51] and other cardiovascular diseases [52,53]. This could suggest that a maladaptive coping mechanism in which psychological distress is expressed through physical symptoms could be a characteristic of TTS.

In line with these findings, the present results confirm that TTS is more associated with poor mental health than other chronic heart diseases. To prove how TTS differs from other heart failures and shows a characteristic psychological pattern, we compared the role of the psychological characteristics in influencing the odd ratio to belong to one of the two clinical conditions. In this sense, our results provide valuable insights into the predictive role of psychological variables, while controlling for sex differences, in discriminating between TTS, AMI, and CG. Our results underline that when comparing individuals with TTS to those with AMI, being female, having lower scores on the MSPSS, having higher scores on anxiety and hostility (assessed by the SCL-90), and having an internal locus of control were significant predictors of being classified in the TTS group. In contrast, when comparing individuals with AMI to the CG, higher scores on the BDI and lower scores on family support scores were associated with an increased likelihood of AMI diagnosis. These findings highlight the importance of further investigating psychological variables in differentiating TTS and AMI and these populations from healthy individuals. Finally, the increased risk of TTS associated with female sex was confirmed [54], underscoring the need for sex-sensitive approaches, especially in TTS.

### Limitation

Some limitations should be highlighted. The main limitation of this study is due to the different proportions of males and females across the groups, especially within the cardiovascular groups. When the analysis was adjusted for sex and other confounding variables, no significant differences were observed. However, the proportion of males in TTS (5.4%) was like that reported in other studies, strengthening the possibility of generalizing the results of the present study to the TTS population. To control for these aspects, we compared two models in multinomial logistic regression, but other studies need to control for the effect of sex when comparing different psychological aspects, with the aim of overcoming the limitations of the sex gap that exists between these two clinical conditions.

Another limitation is the timing of the post-hospital psychological assessment. We do not know whether the results would have been similar if the examination had been conducted earlier or immediately after a clinical episode. The lack of a longitudinal assessment limits the generalizability of the study. However, if the psychological pattern was secondary to the cardiac disease, one might have expected a worse profile in the acute myocardial infarction group because they suffered more sustained myocardial damage. Surely an assessment during hospitalization would provide clear data to support this hypothesis and to define the trajectories and, eventually, the causal relationship between the assessed variables. Moreover, further studies should be included in the psychological assessment, as well as clinical features characterizing the two cardiac conditions, which would help further to explain the differences between TTS and AMI using a combination of medical and psychological features.

## 5. Conclusions

The present study shows that different psychological patterns are associated with different cardiomyopathies. The results of this study could lead to two explanations. First, it is plausible that the cardiac event influences the observed psychological traits. On the other hand, in the light of previous results [8], this psychological feature could be interpreted as a prodrome of a higher susceptibility to stress and a negative reaction to it, which could be considered as a marker of a higher risk of TTS in individuals already predisposed to it.

It is important to consider the psychological aspects of cardiovascular diseases because psychological disorders usually affect the quality of life as well as cognitive profile, which may further contribute to mental health and influence their medical compliance.

The results of this study may have relevant implications for the treatment of patients with TTC, who should receive psychological support to counteract some dysfunctional psychological aspects. The psychological profile of patients with TTS may predispose them to relapse or other cardiac pathologies and may explain some attentional impairments recently highlighted in patients with TTC [55], which may be exacerbated by high levels of anxiety, and which may represent a prodrome of progressive severe cognitive impairment.

## Figures and Tables

**Table 1:** Characteristics of the sample, considering group classification.

	CG	AMI	TTS	χ^2^/F	*p*
N	56	52	56		
Age, mean (SD)	65.6 (12.1)	64.7 (9.5)	71.2 (11.7)	5.30	0.005
Years of Education, mean (SD)	10.9 (4.6)	11.2 (4.2)	8.8 (4.7)	4.96	0.008
Female, n (%)	38 (67.8)	12 (23.1)	53 (94.6)	60.04	0.0001
**Marital Status, n (%)**					
Married	37 (66.2)	39 (75.0)	36 (64.2)		
Single	4 (7.1)	4 (7.7)	1 (1.8)		
Divorced	5 (8.9)	7 (13.5)	1 (1.8)		
Widow	10 (17.8)	2 (3.8)	18 (32.2)		
**Occupational Status, n (%)**					
Employed	26 (46.4)	24 (46.2)	11 (19.6)		
Unemployed	7 (12.5)	4 (7.6)	15 (26.8)		
Retired	23 (41.1)	24 (46.2)	30 (53.6)		
**Cardiac Risk Factor**					
Smoking, n (%)	13 (23.2)	15 (28.9)	6 (10.7)	6.08	0.04
Smoking (n/die), mean (SD)	3.3 (6.4)	6.1 (12.6)	1.5 (4.7)	4.01	0.02
Diabetes, n (%)	9 (16.1)	11 (21.2)	9 (16.1)	<1	0.72
Hypercholesterolemia, n (%)	6 (10.7)	4 (7.7)	4 (7.1)	<1	0.76
Family History of CVD, n (%)	32 (57.4)	36 (69.2)	25 (44.6)	6.69	0.03
Hypertension, n (%)	6 (10.7)	6 (11.5)	1 (1.8)	4.41	0.10
Alcohol Consumption (n/die), mean (SD)	2.6 (3.9)	2.8 (5.2)	1.8 (3.5)	<1	0.47
Body Mass Index, mean (SD)	25.9 (3.9)	26.1 (3.9)	24.2 (4.2)	3.69	0.03
**Clinical measures, mean (SD)**					
SBP	137.1 (21.4)	128.9 (15.6)	145.1 (27.0)	7.15	0.001
DBP	77.2 (12.5)	73.5 (9.6)	75.8 (11.2)	1.51	0.22
HR	71.5 (11.4)	67.8 (11.1)	66.8 (9.9)	2.72	0.07

**Table 2:** Psychological characteristics.

	Health Control Group	Acute Myocardial Infarction	Takotsubo Syndrome	F	*p*
**STAI**					
State Anxiety	36.27 (10.35)	34.71 (11.17)	38.36 (12.42)	1.40	0.25
Trait Anxiety	37.95 (9.58)	36.69 (9.13)	41.02 (11.58) ^a^	2.62	0.07
**SCL-90**					
Somatization	7.63 (5.04)	6.44 (6.01)	12.15 (9.18) ^ab^	10.16	0.001 *
Obsessive-Compulsive	7.32 (5.27)	5.48 (5.44)	8.91 (6.91) ^a^	4.49	0.01 *
Interpersonal Sensitivity	4.65 (4.05)	2.39 (3.13) ^b^	3.71 (4.56)	4.41	0.01
Depression	7.81 (6.28)	6.74 (6.51)	10.19 (7.66)	3.60	0.03
Anxiety	5.10 (4.65)	4.01 (4.62)	5.58 (5.70)	1.35	0.26
Hostility	2.58 (3.41)	1.11 (1.36)	2.21 (2.73) ^a^	4.35	0.01
Phobic Anxiety	1.43 (2.38)	1.52 (2.96)	2.96 (3.86)	4.12	0.02
Paranoid Ideation	3.47 (3.39)	2.48 (2.58)	3.16 (3.60)	1.31	0.27
Psychoticism	2.99 (4.13)	2.17 (2.79)	3.97 (5.25) ^a^	2.46	0.09
Global Score	0.53 (0.36)	0.41 (0.35)	0.65 (0.48) ^a^	5.06	0.007
**MSPSS**					
Family Support	5.68 (1.20)	5.99 (1.31)	6.09 (1.22)	1.65	0.19
Friends Support	5.22 (1.61)	4.63 (1.90)	4.15 (1.92)	4.89	0.009
Other Significant Support	5.96 (1.00)	5.78 (1.15)	5.81 (1.24)	< 1	0.67
**BDI**					
Depression	5.89 (4.50)	6.88 (6.96)	9.46 (9.19)	3.70	0.03
**HLC-S**					
Health Locus Control	34.07 (5.39)	35.29 (8.20)	29.90 (6.94) ^ab^	8.97	0.001

Data were reported considering mean and (standard deviation). STAI: State-Trait Anxiety Inventory; SCL-90: 90-item Symptom Check List; MSPSS: Multidimensional Scale of Perceived Support; BDI: Beck Depression Inventory; HLC-S: Health Locus of Control Scale. * significant statistical value. ^a^: significant difference from Acute Myocardial Infarction; ^b^: significant difference from Health Control Group.

**Table 3:** Results of multinomial logistic regression.

	TTS vs. CG			TTS vs. AMI			AMI vs. CG **		
	Z	*p*	Odds Ratio	Z	*p*	Odds Ratio	Z	*p*	Odds Ratio
Age	−0.11	0.91	0.99	−0.62	0.53	0.98	0.60	0.54	1.02
Sex M-F	2.89 *	0.004	14.62	5.37 *	0.001	490.0	−3.93 **	0.001	0.03
SCL-90 Somatization	−1.88	0.06	0.89	−1.59	0.11	0.89	−0.02	0.98	1.00
SCL-90 Obsessive-Compulsive	0.12	0.90	1.01	−1.05	0.29	0.87	1.20	0.23	1.15
SCL-90 Interpersonal Sensitivity	−3.12	0.002	1.55	−1.19	0.23	1.18	1.91	0.06	1.31
SCL-90 Depression	−0.72	0.47	0.93	−0.55	0.58	1.06	−1.25	0.21	0.87
SCL-90 Anxiety	−3.16	0.002	1.59	−2.83	0.005	1.55	0.25	0.80	1.03
SCL-90 Hostility	−017	0.86	0.97	−1.95	0.05	0.62	1.93	0.05	1.55
SCL-90 Phobic Anxiety	−1.93	0.05	0.78	−0.42	0.67	0.94	−1.25	0.21	0.83
SCL-90 Paranoid Ideation	−0.40	0.69	0.94	−0.37	0.71	0.94	0.05	0.96	1.00
SCL-90 Psychoticism	−2.24	0.02	0.73	−1.50	0.13	0.76	−0.22	0.83	0.96
STAI State Anxiety	−2.28	0.02	0.92	−0.95	0.34	0.96	−1.14	0.25	0.95
STAI Trait Anxiety	−0.45	0.65	0.98	−0.18	0.85	1.01	−0.62	0.52	0.97
BDI Depression	−0.84	0.40	0.93	1.58	0.11	1.16	−2.14	0.03	0.80
Health Locus of Control	−1.99	0.04	1.11	−2.55	0.01	1.15	−0.83	0.41	0.96
MSPSS Family Support	−1.68	0.09	0.58	1.05	0.29	1.48	−2.48	0.01	0.40
MSPSS Friend Support	3.05	0.002	1.75	2.13	0.03	1.58	0.48	0.62	1.10
MSPSS Other Significant Support	1.13	0.26	1.47	−2.13	0.03	0.45	3.09	0.002	3.26

* Reference level was set in TTS/Female. ** Reference level was set in AMI/Female. STAI: State-Trait Anxiety Inventory; SCL-90: 90-item Symptom Check List; MSPSS: Multidimensional Scale of Perceived Support; BDI: Beck Depression Inventory; HLC-S: Health Locus of Control Scale.

## Data Availability

Data can be made available by contacting the corresponding author.
